# Force Reversal During Systolic–Diastolic Transition Provides Incremental Prognostic Value over LVEF for Heart Failure After STEMI

**DOI:** 10.3390/jcm14227978

**Published:** 2025-11-11

**Authors:** Yumeng Sun, Xinyu Wu, Lu Li, Tingting Li, Zhenjia Wang, Wei Yu

**Affiliations:** Department of Radiology, Anzhen Hospital, Affiliated to Capital Medical University, 2 Anzhen Road, Chao Yang District, Beijing 100029, China; sym1997928@163.com (Y.S.); zhuzongsz@163.com (X.W.); 13033420084@163.com (L.L.); letdxhc@163.com (T.L.); dr_zhenjiawang@163.com (Z.W.)

**Keywords:** cardiac magnetic resonance, hemodynamic forces, myocardial infarction, infarct location, infarct size

## Abstract

**Background:** Left ventricular hemodynamic forces (LV HDFs) are altered in myocardial dysfunction. While infarct location and size influence post-ST-segment elevation myocardial infarction (STEMI) remodeling, their specific effects on HDFs remain unclear. This study investigated how infarct location and size impact left ventricular HDFs and assessed HDFs’ prognostic value for predicting subsequent heart failure (HF) in STEMI patients. **Methods:** In this retrospective study, 275 STEMI patients underwent cardiac magnetic resonance (CMR) 3–7 days after primary percutaneous coronary intervention. HDFs were derived from routine CMR cine images. Patients were stratified by infarct location (anterior vs. non-anterior) and median infarct size (IS). Key parameters—apical–basal (A-B) and lateral-septal (L-S) forces, their ratio, force direction angle (φ), and force reversal during systolic–diastolic transition—were compared. The primary endpoint was new-onset congestive HF during follow-up. **Results:** Compared to non-anterior STEMI, anterior STEMI showed significantly impaired A-B and L-S HDFs throughout the cardiac cycle (all *p* < 0.05) and was independently associated with force reversal (OR 2.31, 95% CI: 1.05–5.07). Larger IS correlated with reduced A-B HDFs and altered force distribution (increased L-S/A-B ratio, decreased φ). Force reversal predicted HF (HR: 2.10, 95% CI: 1.22–3.62) and provided incremental prognostic value beyond left ventricular ejection fraction (LVEF) alone (C-statistic: LVEF 0.680 vs. LVEF + force reversal 0.770, *p* = 0.034). **Conclusions:** Anterior infarction causes global HDF impairment and force reversal, while larger IS primarily reduces longitudinal forces and disrupts force distribution. Force reversal predicts subsequent HF and enhances prognostic value beyond LVEF.

## 1. Introduction

The segmental wall mechanics interact dynamically with left ventricular (LV) fluid dynamics during filling and ejection [1,2,3,4,5,6]. Blood flow analysis offers unique physiological insights beyond conventional imaging, as it reflects intraventricular pressure gradients (IVPGs) generated by the myocardium–valve–vessel interplay. Hemodynamic forces (HDFs) [7], defined as the volumetric integration of IVPGs, provide a rigorous framework to quantify these interactions. HDFs capture the spatiotemporal evolution of pressure gradients driven by blood-tissue motion, serving as a fluid dynamic counterpart to deformation imaging.

Previous investigations into LV HDFs have primarily utilized echocardiography or 4D flow cardiac magnetic resonance (CMR). However, these techniques face limitations in clinical applicability and adoption due to technical complexity and limited availability [8]. Recent advances have enabled the derivation of HDFs from CMR cine images through first-principles modeling [9], with validation against 4D flow CMR [10]. This approach eliminates the need for direct velocity measurements and enhances clinical utility, as cine imaging is a standard sequence that is routinely acquired in conventional CMR protocols.

Alterations in HDFs are closely associated with myocardial dysfunction [5,11,12,13]. Notably, Domenico Filomena et al. recently demonstrated that misalignment of LV HDFs correlates with adverse LV remodeling following ST-segment elevation myocardial infarction (STEMI) [11]. While infarct characteristics (e.g., size and location) are established determinants of post-STEMI remodeling and clinical outcomes [11], their specific relationship with LV HDFs remains incompletely characterized. Numerous parameters derived from CMR, such as late gadolinium enhancement (LGE), infarct size (IS), microvascular obstruction (MVO), global longitudinal strain (GLS), T2 values in non-infarcted myocardium, and left ventricular ejection fraction (LVEF), have been established as significant prognostic markers [11,14]. Early research has demonstrated that HDFs are profoundly altered in conditions such as heart failure (HF) and myocardial infarction (MI), and may modulate the response to HF therapy [3,11,15], highlighting their potential in detecting cardiac dysfunction and predicting clinical outcomes. Against this backdrop, this study had two primary objectives: first, to investigate the correlation between infarct characteristics and LV HDF alterations in patients with STEMI using a clinically feasible CMR cine-based approach; and second, to assess the prognostic significance of LV HDFs for predicting subsequent HF.

## 2. Materials and Methods

This retrospective study included consecutive STEMI patients from January 2019 to January 2023 who met the following criteria: (1) successful revascularization via percutaneous coronary intervention (PCI); (2) completion of CMR within 3–7 days post-STEMI; and (3) presence of sinus rhythm on electrocardiogram (ECG). CMR image quality was graded on a 4-point scale (1: nondiagnostic; 2: poor; 3: good; and 4: excellent) [16]. Only CMR images with image quality > 2 were used. Exclusion criteria comprised prior MI or revascularization, major comorbidities (e.g., cardiomyopathies, valvular disease, or arrhythmias such as atrial fibrillation/bundle branch block), and inadequate CMR quality (score ≤ 2). Demographic and clinical characteristics were systematically recorded. The institutional ethics committee approved the study protocol. Written informed consent was waived due to the retrospective nature of the study.

### 2.1. CMR Acquisition

All examinations were conducted on 3T MRI systems (GE Healthcare’s MR750W, Waukesha, WI, USA, Philips’ Ingenia CX, Best, The Netherlands). The CMR protocol utilized a balanced steady-state free precession (bSSFP) sequence for cine imaging, acquiring full short-axis coverage (8 mm slices) from apex to base, plus standard long-axis views (2-, 3-, and 4-chamber; 5 mm slices). For the GE system, the parameters were as follows: repetition time (TR), 3.6 ms; echo time (TE), 1.4 ms; flip angle, 60°; field of view (FOV), 380 × 380 mm^2^; and temporal resolution, 86 ms. For the Philips system, the parameters were as follows: TR, 3.0 ms; TE, 1.5 ms; flip angle, 45°; FOV, 270 × 270 mm^2^; and temporal resolution, 50 ms. LGE imaging was performed using phase-sensitive inversion recovery 10–15 min post-injection of 0.2 mmol/kg Magnevist contrast agent, maintaining identical imaging planes to the cine acquisitions. Acquisition parameters for LGE were as follows: on the GE system—TR, 6.2 ms; TE, 2.9 ms; flip angle, 25°; FOV, 380 × 380 mm^2^; temporal resolution, 149 ms; and on the Philips system—TR, 6.1 ms; TE, 3.0 ms; flip angle, 25°; FOV, 350 × 350 mm^2^; temporal resolution, 154 ms. Complete acquisition parameters were documented in a previous study [17].

### 2.2. CMR Image Analysis

Cardiac functional parameters, including LVEF, end-diastolic volume (EDV), and end-systolic volume (ESV), were quantified using CVI42 software (Circle Cardiovascular Imaging, Calgary, AB, Canada). Post-contrast analysis automatically detected LGE as myocardial regions exceeding 5 standard deviations (SDs) of remote myocardial signal intensity. MVO, characterized by hypoenhanced areas within hyperintense myocardium, was incorporated into LGE measurements when present. Infarct localization was determined by LGE distribution, with anterior infarction diagnosed when involving any of these segments: basal anteroseptal, mid-anterior, mid-anteroseptal, or apical anterior [11]. Strain and HDFs analysis utilized QStrain (Medis Medical Imaging, Leiden, The Netherlands) with manual endocardial contour tracing on end-systolic/diastolic frames, deriving GLS and GCS from standard long-axis cine views.

In the context of the endocardial border tracking used for GLS calculation, HDFs can be obtained by measuring the aortic and mitral diameters using a previously validated mathematical model [10]. The feature-tracking strain methodology applied for HDF estimation was previously validated in a study investigating HDF parameters associated with adverse left ventricular remodeling following STEMI [11]. We measured aortic and mitral valve diameters from three-chamber views. These measurements enabled the generation of the following: (1) time-varying HDF curves for both A-B and lateral-septal L-S directions, and (2) polar histograms depicting LV HDF distribution. To ensure size-independent comparison, HDF values were normalized to LV volume and expressed as a percentage of gravitational acceleration. This dimensionless representation essentially reflects the integrated intracavitary pressure gradients.

**Figure 1:**
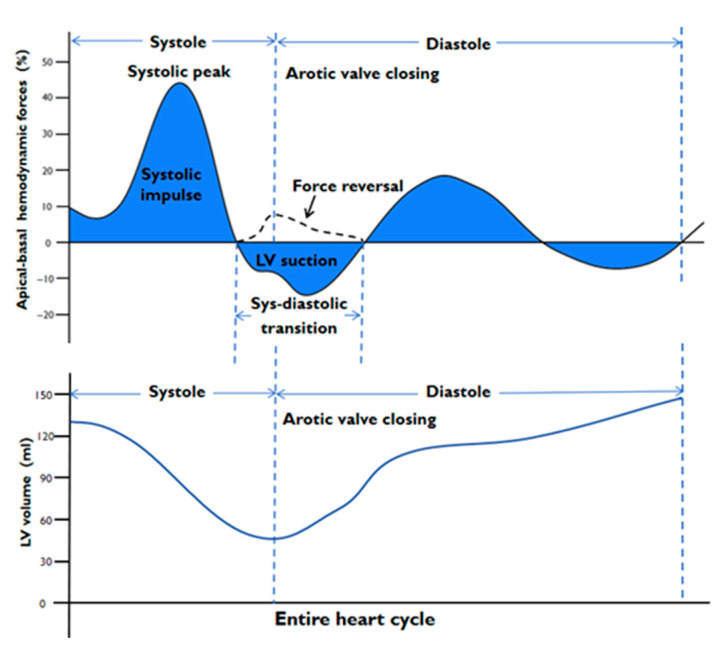
Schematic of left ventricular hemodynamic forces during a cardiac cycle. The top curve represents the apex–base component of the HDFs. If the HDFs are directed from apex to base (apical pressures are higher), it is depicted as a positive wave. If the HDFs are directed from base to apex (basal pressures are higher), it is depicted as a negative wave. Key phases and parameters are demarcated: systole (from aortic valve opening to closing), diastole (from aortic valve closing to the next cycle), and the systolic–diastolic transition phase. The systolic–diastolic transition phase is characterized by a brief but negative impulse, which is a hallmark of efficient ventricular relaxation and contributes to early diastolic LV filling. Systolic peak: Maximum value of apex–base HDFs during systole. Systolic impulse: The mean amplitude of the apex–base HDFs during the positive systolic phase, when the force has an apex–base direction. LV suction: The mean amplitude of the apex–base HDFs across the diastolic phases, when the force is negative (base–apex direction), encompasses end-systole and isovolumic relaxation. Force reversal refers to a temporary reversal of HDFs from base to apex (below the zero line) to apex–base (above the zero line) in systolic–diastolic transition. The lower curve represents schematic changes in LV volume during the cardiac cycle. HDFs: hemodynamic forces; LV: left ventricular.

If the HDFs are directed from apex to base (apical pressures are higher), they are depicted as a positive wave. If the HDFs are directed from base to apex (basal pressures are higher), it is depicted as a negative wave. Prior research has detailed the key parameters of HDFs [8,18], which include the following: Root mean square (RMS): Reflects overall force amplitude, incorporating both positive and negative values. Systolic peak: Maximum value of apex–base HDFs during systole. Systolic impulse: The mean amplitude of the apex–base HDFs during the positive systolic phase, when the force has an apex–base direction. LV suction: The mean amplitude of the apex–base HDFs across the diastolic phases, when the force is negative (base–apex direction), encompasses end-systole and isovolumic relaxation. Force reversal referred to a temporary reversal of HDFs from base to apex (below the zero line) to apex–base (above the zero line) in the systolic–diastolic transition [5]. Figure 1 illustrates the typical A-B HDF components over the cardiac cycle and phase definitions. L–S/A–B HDFs ratio (%): Evaluates directional distribution by comparing L-S and A-B HDFs. HDF’s angle [φ (°)]: Represents the dominant force direction over a defined period in polar coordinates, with φ varying from 0° to 360°. A value of 0° corresponds to a transverse distribution, while 90° indicates a longitudinal distribution. We calculated the main HDF parameters in the following phases: entire heart cycle, systole, systolic–diastolic transition, and diastole.

### 2.3. Follow-Up

During the clinical follow-up, evaluations were conducted at six-month intervals through telephone contact or case review. New-onset congestive HF served as the primary endpoint, designated by a first occurrence of cardiac decompensation that required diuretic therapy [19]. The follow-up period extended to early May 2024, and the endpoint for each participant was marked by either the final study date or the ascertainment of loss to follow-up.

### 2.4. Statistical Analysis

Normally distributed continuous data were reported as mean ± SD and were analyzed with Student’s *t*-test, while non-normally distributed data were presented as median (interquartile range, IQR) and assessed using the Mann–Whitney U test. For categorical variables, frequencies (percentages) were used, with comparisons made via the chi-square test or Fisher’s exact test, as appropriate. The STEMI population was stratified by infarct location into anterior and non-anterior STEMI groups, as well as by IS into subgroups below and above the median value. Routine CMR and HDF parameters were compared across these groups. To identify risk factors linked to force reversal in systolic–diastolic transition, logistic regression analysis was employed. Cox proportional hazards regression analyses were conducted to investigate associations with HF. Only variables with a *p*-value < 0.05 were included in further multivariable regression calculations. We used the variance inflation factor (VIF) to detect multicollinearity among variables in multivariate analysis. The results of the multicollinearity analysis are presented in Table S1. In order to avoid collinearity, GCS and LVEF were tested separately in a multivariable regression. The assessment and comparison of model performance were conducted by employing the Harrell C-statistic. C-statistic results were compared using the nonparametric method previously described by De Long et al. [20]. The calibration curve was used to assess the model’s calibration. Spearman’s correlation analysis was used to determine correlations between IS and HDF variables. Reproducibility of HDF parameters was evaluated through the intraclass correlation coefficient (ICC) with 95% confidence intervals (CI), where ICC < 0.5 indicated poor agreement and ICC > 0.75 denoted good agreement for both intraobserver and interobserver assessments [21]. Statistical significance was set at a two-tailed *p* < 0.05. IBM SPSS Statistics 25.0 (Armonk, NY, USA) and R 4.4.3 (The R Foundation, Vienna, Austria) were used for statistical analyses.

## 3. Results

### 3.1. Patients’ Baseline and Clinical Characteristics

**Figure 2:**
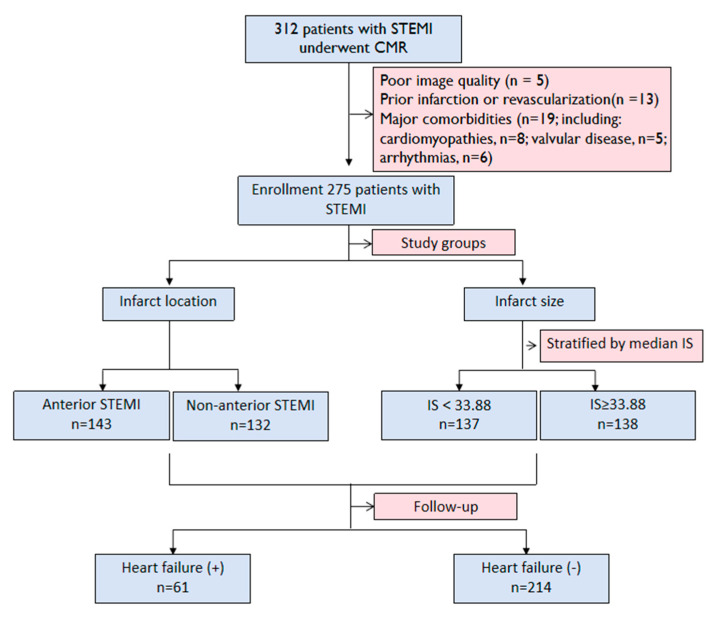
The flowchart of our study. STEMI, ST-segment elevation myocardial infarction; CMR, cardiac magnetic resonance; IS, infarct size.

From January 2019 to January 2023, a total of 312 STEMI patients who underwent CMR were enrolled. After excluding patients with major comorbidities (n = 19), poor image quality (n = 5), and prior infarction or revascularization (n = 13), 275 patients were included in the final analysis (Figure 2). Among the 275 patients, 61 developed HF. The mean follow-up time was 35 (IQR: 24–48) months. The baseline and clinical characteristics of the study population are presented in Table 1. The median age of the cohort was 58 years (IQR: 49–67), and 229 patients (83%) were male.

### 3.2. The Association Between Left Ventricular Hemodynamic Forces and Infarct Location

**Figure 3:**
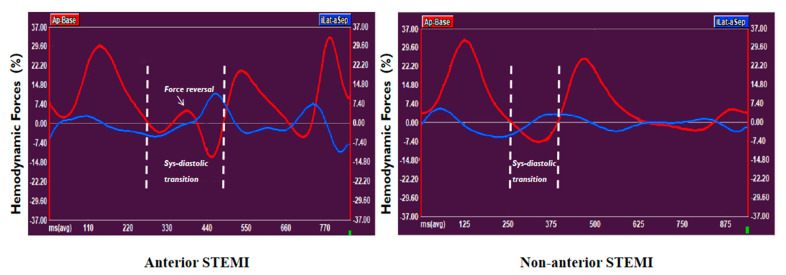
The relationship between infarct location and force reversal in the systolic–diastolic transition. On the left, anterior STEMI exhibits force reversal in the systolic–diastolic transition; on the right, non-anterior STEMI shows no force reversal in the same phase. STEMI, ST-segment elevation myocardial infarction.

The STEMI population was divided into anterior STEMI (52%) and non-anterior STEMI (48%) based on infarct location (Table 2). Patients with anterior STEMI exhibited a larger IS (37.2% vs. 30.5%; *p* < 0.001), as well as reduced GLS and LVEF (GLS: −11.6 vs. −18.7, *p* < 0.001; LVEF: 47.9% vs. 52.8%, *p* = 0.001, respectively). Throughout the entire heart cycle, systolic–diastolic transition, and diastole, anterior STEMI was associated with significantly lower A-B HDFs compared to non-anterior STEMI (all *p* < 0.05). Similarly, L–S HDFs were significantly reduced in anterior STEMI across all cardiac phases (all *p* < 0.05). Furthermore, anterior STEMI showed a higher prevalence of force reversal during the systolic–diastolic transition than non-anterior STEMI (25% vs. 13%, *p* = 0.01, Table 2; Figure 3). Although anterior STEMI was associated with numerically weaker LV suction during the systolic–diastolic transition compared to non-anterior STEMI, the difference did not reach statistical significance (−4.8 vs. −5.7, *p* = 0.056). Notably, no significant differences were observed in the L–S/A–B HDFs ratio and HDFs angle φ between the two groups across any cardiac phase (all *p* > 0.05) (Table 2).

### 3.3. The Association Between Left Ventricular Hemodynamic Forces and Infarct Size

The STEMI population was further stratified into two groups based on whether the IS was below or above the median value (Table 3). Patients with above-median IS had a higher prevalence of anterior STEMI, more extensive MVO, and more severe LV dysfunction. Compared to those with below-median IS, patients with larger infarcts showed significantly more impaired LV GLS and GCS (all *p* < 0.001). LVEF was also significantly lower in the above-median IS group (46.6% vs. 55.8%, *p* < 0.001). A–B HDFs were significantly reduced in patients with above-median IS throughout the entire heart cycle, systole, systolic–diastolic transition, and diastole (all *p* < 0.05). Both systolic impulse and systolic peak were notably lower in this group (systolic impulse: 10.7 vs. 14.2, *p* < 0.001; systolic peak: 22.1 vs. 26.7, *p* = 0.003). LV suction during the systolic–diastolic transition was significantly weaker in patients with above-median IS (−4.6 vs. −5.7, *p* = 0.006). Although force reversal was more frequent in the above-median IS group (22% vs. 17%), this difference was not statistically significant (*p* = 0.292).

Additionally, the above-median IS group exhibited a significantly elevated L–S/A–B HDF ratio and a reduced HDF angle φ during the entire heart cycle and systole (all *p* < 0.05; Table 3). Spearman correlation analysis revealed modest associations between larger IS and impaired HDF parameters, including reduced A–B HDFs (r = −0.22 to −0.24), decreased HDF angle φ (r = −0.23 to −0.29), and increased L–S/A–B ratios (r = 0.24 to 0.25) across all cardiac phases (all *p* < 0.01; Table S2).

### 3.4. Univariable and Multivariable Predictors of Force Reversal in Systolic–Diastolic Transition

The univariable logistic regression identified five significant predictors of force reversal in systolic–diastolic transition (all *p* < 0.05). The strongest predictors were anterior STEMI (OR 2.27, 95% CI 1.20–4.29), GLS (OR 1.05, 95% CI 1.00–1.10), GCS (OR 1.04, 95% CI 1.00–1.08), LVEF (OR 0.97, 95% CI 0.95–0.99), and MVO (OR 1.06, 95% CI 1.00–1.12). Notably, IS showed no significant association with force reversal in systolic–diastolic transition (OR 1.01, 95% CI 0.99–1.03, *p* = 0.065). Demographic factors showed no significant association (all *p* > 0.1). After multivariable adjustment for IS, GLS, GCS, LVEF, and MVO, anterior STEMI remained independently associated with force reversal in systolic–diastolic transition (Table 4).

### 3.5. Incremental Value of Force Reversal for Predicting HF in Patients with STEMI

In the univariable Cox regression analyses, force reversal, anterior STEMI, GCS, GLS, LVEF, IS, and MVO were significantly associated with HF following STEMI (all *p* < 0.05). After multivariable adjustment for force reversal, anterior STEMI, GCS, LVEF, and MVO, GLS and IS remained independently associated with HF (GLS: HR 1.09, 95% CI, 1.06–1.13, *p* < 0.001; IS: HR 1.02, 95% CI: 1.02–1.05, *p* < 0.001, Table 5). Table 6 presents the prognostic performance of models for predicting HF in patients with STEMI. The inclusion of the force reversal in addition to LVEF for the prediction of HF resulted in a significant increase in the C-statistics (Model 1: LVEF alone, C-statistics: 0.680; 95% CI: 0.610–0.747 vs. Model 6: Force reversal + LVEF, C-statistics: 0.770; 95% CI: 0.721–0.819; *p* = 0.034). The results demonstrate the additive prognostic value of the force reversal over LVEF for HF. Figure S1 shows the calibration curve of Model 6 for predicting HF with a follow-up of 1 year. While adding force reversal to IS, GLS, and anterior STEMI increased the C-statistic for HF prediction, the increase was not statistically significant (Table 6).

### 3.6. Reproducibility Evaluation

In a cohort of 30 patients, the intra- and inter-observer variability in HDF measurements was assessed. Two independent examiners conducted blinded evaluations of the same examination, with one examiner repeating the measurements after a one-week interval without knowledge of prior results. Our findings demonstrated poor-to-excellent agreement for all HDF parameters, both within and between observers, which is consistent with previously reported results [22,23]. Intraclass correlation coefficients are reported in Table S3.

## 4. Discussion

In this study, we investigated the impact of infarct characteristics on left ventricular HDFs and initially explored the prognostic value of HDFs for HF in STEMI patients. Our key findings demonstrate the following: (1) Anterior STEMI is associated with significantly impaired multidirectional HDFs (A-B and L-S) throughout the cardiac cycle, whereas IS correlates predominantly with reduced A-B HDFs and altered force distribution patterns; (2) Anterior STEMI, but not IS, is independently associated with force reversal in the systolic–diastolic transition; and (3) Force reversal emerged as a significant predictor of HF after STEMI, providing incremental prognostic value beyond LVEF.

Efficient ventricular contraction generates substantial HDFs, which directly facilitate systolic ejection. Following STEMI, reduced HDFs may stem either from loss of contractile myocardium or from abnormal spatiotemporal contraction patterns. Such abnormalities can impair mechanical and electrical synchrony, weakening the resultant thrust toward the LV outflow tract. In our study, the observed reduction in both A-B and L-S (latero-septal) HDFs in anterior STEMI patients suggests global hemodynamic impairment, likely reflecting the more extensive ventricular involvement characteristic of anterior wall infarction. This finding aligns with previous studies that demonstrated worse mechanical dysfunction in anterior STEMI [24,25]. Importantly, our data extend these observations by quantifying the temporal persistence of these abnormalities across all cardiac phases.

Under normal physiological conditions, HDFs are oriented predominantly along the longitudinal (base–apex) axis. This alignment supports blood flow following the heart’s intrinsic base-to-apex pathway, thereby reducing energy expenditure during stroke volume generation. Although the three-dimensional anatomy of the heart inevitably introduces minor transversal (latero-septal) components, substantial transversal HDFs arise only when synchrony in myocardial segmental deformation is disrupted. Such dyssynchrony produces abnormally directed pressure gradients. If the magnitude of transversal HDFs becomes comparable to that of longitudinal components—as indicated by an increased L-S/A-B HDFs ratio and a decreased HDFs angle φ—these forces reflect mechanical inefficiency. In this scenario, different myocardial regions exert opposing forces through the incompressible blood volume, consuming energy without contributing effectively to cardiac ejection or filling [8]. Large infarcts disrupt coordinated segmental deformation, with compensatory hypercontractility of non-infarcted regions generating aberrant L-S pressure gradients against passive stretching of infarcted areas. This increases L-S HDFs, while the A-B HDFs are relatively diminished due to impaired global ejection, elevating the L-S/A-B HDFs ratio. Reduced HDF’s angle φ reflects “counterproductive” forces—where healthy segments push against infarcted myocardium through the incompressible blood pool, dissipating energy as internal work rather than effective ejection. This aligns with the “inefficient work” hypothesis. The more pronounced difference during systole may stem from exacerbated mechanical dyssynchrony under high-pressure loading, whereas diastolic discrepancies could arise from heterogeneous relaxation.

During the systolic–diastolic transition, the LV generates low pressure at the apex through shape changes and elastic recoil (the “suction effect”), establishing a base-to-apex pressure gradient that facilitates early diastolic filling. Following STEMI, anterior wall infarction may directly damage the apical myocardium (the “engine” of the suction effect), leading to reduced stored elastic potential energy, disruption of apical vortex dynamics, and the consequent attenuation or even loss of LV suction [26]. This results in transiently higher pressure at the apex compared to the base. The reversal of this pressure gradient (force reversal) impairs early diastolic filling. The detection of force reversal may provide an early hemodynamic marker for severely impaired LV suction before overt diastolic dysfunction becomes apparent. In our study, force reversal emerged as a predictor of HF following STEMI, providing incremental prognostic value beyond LVEF. This may facilitate the diagnosis of heart failure with preserved ejection fraction (HFpEF) or HF with mildly reduced EF. It is well-established that LVEF serves as a cornerstone for assessing cardiac pump function but possesses inherent limitations in identifying intrinsic myocardial mechanical abnormalities, particularly during diastole. Many patients, especially those with potential HFpEF, may present with normal or near-normal LVEF and yet already exhibit significant diastolic dysfunction and clinical symptoms. As early as 2019, a study by Tomas Lapinskas et al. published in JACC demonstrated that there was no significant difference in LVEF or strain between healthy volunteers and HFpEF patients, whereas the HFpEF group exhibited significantly abnormal HDFs compared to healthy volunteers [3]. Our findings suggest that force reversal can capture early myocardial functional impairments that remain undetected by LVEF alone. Consequently, integrating force reversal with LVEF assessment promises to identify patients with “normal” LVEF who are at high risk of HF and who might benefit from more aggressive follow-up and therapy.

Recent evidence indicates that after adjusting for clinical and therapeutic profiles, one-year outcomes are largely comparable between patients with STEMI and non-ST-elevation myocardial infarction (NSTEMI) [27]. This suggests that specific pathophysiological alterations—such as infarct location and size—and treatment patterns, rather than the broad diagnostic category itself, may be the primary determinants of long-term prognosis. LV HDF analysis can provide insights into cardiac pathophysiological alterations that cannot be obtained with conventional cardiovascular imaging. Our findings reveal that within the STEMI population, infarct location and size exert significantly distinct impacts on LV hemodynamics. These hemodynamic aberrations likely contribute to an elevated risk of HF, underscoring the clinical relevance of our assessment.

### Limitations of the Study

Several limitations merit consideration. First, the single-center, retrospective nature of our study may limit the generalizability of the results and introduce potential selection biases inherent to retrospective analyses. Second, CMR was performed 3–7 days after PCI. During this acute-to-subacute phase, LGE may not solely reflect myocardial infarction but could also include areas of edema within the peri-infarct zone. This overlap may lead to overestimation of the true IS and potentially confound the association between infarct characteristics and hemodynamic alterations. Finally, as an initial investigation into the association between infarct characteristics and LV hemodynamic forces, this study did not incorporate longitudinal outcome assessments. Future prospective studies are warranted to validate the prognostic significance of these hemodynamic parameters and to explore their potential role in clinical decision-making.

## 5. Conclusions

Preliminary findings suggest distinct hemodynamic impacts of infarct location and size in STEMI. Anterior infarction may cause global HDF impairment and is independently associated with force reversal in systolic–diastolic transition, while larger IS primarily reduces longitudinal (apex–base) forces and disrupts force distribution. Force reversal predicted HF and may have incremental value over LVEF. These hypotheses merit further prospective validation.

## Figures and Tables

**Table 1:** Patient baseline and clinical characteristics.

	STEMI Patients N = 275
Age, median (IQR), years	58 (49–67)
Male, %	229 (83%)
Cardiovascular risk factors	
Smoking, %	174 (63%)
Hypertension, %	166 (60%)
Hyperlipoproteinemia, %	255 (92%)
Diabetes mellitus, %	88 (32%)
BMI, median (IQR), kg/m^2^	26.0 (24.2–28.3)
SBP, median (IQR), mm Hg	122 (110–132)
DBP, median (IQR), mm Hg	75 (68–80)
Heart rate, median (IQR), beats/min	76 (70–84)
Anterior STEMI, n (%)	143 (52%)
Stent implanted	228 (82%)
Infarct-related artery	
Left anterior descending	142 (52%)
Left circumflex	33 (12%)
Right coronary	100 (36%)
Number of diseased vessels	
1	107 (39%)
2	73 (27%)
3	95 (34%)
Killip class on admission	
1	202 (74%)
2	56 (20%)
3	5 (2%)
4	12 (4%)
TIMI flow grade before PCI	
0	175 (64%)
1	13 (5%)
2	29 (10%)
3	58 (21%)
TIMI flow grade post-PCI	
0	2 (1%)
1	2 (1%)
2	7 (2%)
3	264 (96%)

Abbreviations: IQR, interquartile range; BMI, body mass index; SBP, systolic pressure at presentation; DBP, diastolic pressure at presentation; STEMI, ST-segment elevation myocardial infarction; TIMI, thrombolysis in myocardial infarction; and PCI, percutaneous coronary intervention.

**Table 2:** Comparison of CMR findings between patients with anterior and non-anterior STEMI.

Parameter	STEMI Patients N = 275	Anterior STEMI N = 143	Non-Anterior STEMI N = 132	*p* Value
GLS (%)	−15.2 (−19.8, −10.8)	−11.6 (−15.3, −8.0)	−18.7 (−22.0, −15.4)	<0.001
GCS (%)	−24.1 ± 7.8	−23.2 ± 8.1	−25.0 ± 7.4	0.062
EDV (mL)	139.1 (112.4, 167.5)	143.2 (115.5, 172.2)	134.4 (110.8, 164.0)	0.052
ESV (mL)	67.9 (45.9, 92.5)	71.6 (49.5, 103.6)	63.6 (45.0, 82.2)	0.007
LVEF (%)	50.5 ± 12.1	47.9 ± 13.1	52.8 ± 10.7	0.001
IS (%)	34.0 ± 15.3	37.2 ± 16.1	30.5 ± 13.6	<0.001
MVO (%)	1.4 (0, 5.0)	1.5 (0.2, 5.7)	1.3 (0, 4.9)	0.232
**HDFs: entire heart cycle**				
A-B (%) (RMS)	11.2 (8.1, 15.5)	10.4 (7.3, 14.8)	11.8 (8.8, 16.2)	0.032
L-S (%) (RMS)	2.7 (2.0, 3.4)	2.5 (1.9, 3.2)	2.9 (2.2, 3.7)	0.003
L-S/A-B HDFs ratio (%)	25.0 (19.1, 29.4)	24.7 (19.0, 29.2)	25.3 (19.1, 29.4)	0.611
Angle φ (°)	70 (67, 74)	70 (67, 74)	71 (67, 74)	0.533
**HDFs: systole**				
A-B (%) (RMS)	13.1 (8.7, 18.6)	12.8 (8.5, 18.0)	13.2 (9.0, 19.3)	0.400
L-S (%) (RMS)	2.4 (1.8, 3.2)	2.3 (1.6, 3.1)	2.4 (1.9, 3.3)	0.046
L-S/A-B HDFs ratio (%)	19.0 (13.9, 25.4)	18.8 (13.9, 24.3)	19.3 (13.9, 26.4)	0.608
Angle φ (°)	74 (69, 77)	74 (70, 77)	73 (69, 76)	0.249
Systolic impulse (%)	12.2 (8.2, 18.2)	12.0 (8.0, 17.0)	12.9 (8.4, 18.8)	0.312
Systolic peak (%)	24.4 (16.9, 35.4)	24.2 (17.0, 33.9)	25.5 (16.9, 35.4)	0.490
**HDFs: systolic–diastolic transition**				
A-B (%) (RMS)	6.6 (4.4, 10.2)	6.0 (3.9, 9.5)	7.2 (4.6, 10.8)	0.049
L-S (%) (RMS)	3.5 (2.3, 4.8)	3.0 (1.8, 3.9)	3.9 (2.9, 5.6)	<0.001
L-S/A-B HDFs ratio (%)	52.0 (36.5, 68.6)	49.6 (33.4, 66.5)	53.4 (39.6, 72.0)	0.073
Angle φ (°)	60 (54, 66)	61 (56, 67)	60 (55, 62)	0.288
LV suction (%)	−5.1 (−8.1, −3.3)	−4.8 (−7.4, −3.1)	−5.7 (−8.8, −3.7)	0.056
Force reversal (%)	53 (19%)	36 (25%)	17 (13%)	0.01
**HDFs: diastole**				
A-B (%) (RMS)	8.0 (5.5, 10.9)	7.3 (5.0, 10.2)	8.4 (6.3, 11.6)	0.008
L-S (%) (RMS)	2.7 (1.9, 3.7)	2.6 (1.8, 3.1)	3.0 (2.1, 4.0)	<0.001
L-S/A-B HDFs ratio (%)	34.9 (25.8, 44.5)	35.9 (25.6, 43.0)	34.5 (26.0, 46.4)	0.681
Angle φ (°)	68 (63, 72)	68 (63, 72)	67 (63, 72)	0.647

Abbreviations: CMR, cardiac magnetic resonance; STEMI, ST-segment elevation myocardial infarction; GLS, global longitudinal strain; GCS, global circumferential strain; EDV, end-diastolic volume; ESV, end-systolic volume; LVEF, left ventricular ejection fraction; IS, infarct size; MVO, microvascular obstruction; HDFs, hemodynamic forces; RMS, root mean square; A-B, apex–base; and L-S, latero-septal.

**Table 3:** Comparison of CMR findings between patients with infarct size below and above the median.

Parameter	STEMI Patients N = 275	IS < 33.88 N = 137	IS ≥ 33.88 N = 138	*p* Value
GLS (%)	−15.2 (−19.8, −10.8)	−17.4 (−21.7, −13.4)	−12.6 (−17.2, −8.3)	<0.001
GCS (%)	−24.1 ± 7.8	−26.7 ± 7.5	−21.5 ± 7.3	<0.001
LVEF (%)	139.1 (112.4, 167.5)	55.8 (48.6, 62.9)	46.6 (37.2, 54.3)	<0.001
EDV (mL)	67.9 (45.9, 92.5)	132.1 (105.6, 154.7)	146.5 (117.5, 172.6)	0.001
ESV (mL)	50.5 ± 12.1	55.9 (42.8, 72.6)	77.3 (55.5, 102.9)	<0.001
IS (%)	34.0 ± 15.3	24.1 (15.4, 28.7)	43.8 (38.3, 51.1)	<0.001
MVO (%)	1.4 (0, 5.0)	0.3 (0, 2.4)	3.6 (0.7, 8.1)	<0.001
Anterior STEMI	143 (52%)	60 (43.8%)	83 (60.1%)	0.007
**HDFs: entire heart cycle**				
A-B (%) (RMS)	11.2 (8.1, 15.5)	12.4 (8.9, 16.7)	10.3 (7.3, 13.5)	0.002
L-S (%) (RMS)	2.7 (2.0, 3.4)	2.8 (2.1, 3.8)	2.6 (1.9, 3.3)	0.073
L-S/A-B HDFs ratio (%)	25.0 (19.1, 29.4)	23.9 (18.6, 28.0)	26.0 (20.7, 30.3)	0.025
Angle φ (°)	70 (67, 74)	71 (68, 74)	69 (66, 72)	0.003
**HDFs: systole**				
A-B (%) (RMS)	13.1 (8.7, 18.6)	14.0 (9.9, 21.3)	11.8 (7.9, 15.6)	0.002
L-S (%) (RMS)	2.4 (1.8, 3.2)	2.4 (1.8, 3.3)	2.3 (1.7, 3.2)	0.565
L-S/A-B HDFs ratio (%)	19.0 (13.9, 25.4)	17.5 (12.9, 22.7)	20.1 (15.6, 27.0)	0.002
Angle φ (°)	74 (69, 77)	75 (71, 78)	73 (68, 76)	0.001
Systolic impulse (%)	12.2 (8.2, 18.2)	14.2 (9.8, 20.7)	10.7 (7.4, 15.5)	<0.001
Systolic peak (%)	24.4 (16.9, 35.4)	26.7 (18.7, 40.6)	22.1 (15.1, 30.6)	0.003
**HDFs: systolic–diastolic transition**				
A-B (%) (RMS)	6.6 (4.4, 10.2)	7.5 (5.0, 11.3)	5.7 (3.8, 9.0)	0.003
L-S (%) (RMS)	3.5 (2.3, 4.8)	3.9 (2.6, 5.4)	3.2 (2.0, 4.4)	0.002
L-S/A-B HDFs ratio (%)	52.0 (36.5, 68.6)	51.3 (36.4, 68.7)	51.0 (34.1, 66.5)	0.840
Angle φ (°)	60 (54, 66)	60 (54, 66)	59 (54, 66)	0.997
LV suction (%)	−5.1 (−8.1, −3.3)	−5.7 (−8.8, −3.9)	−4.6 (−7.1, −3.0)	0.006
Force reversal (%)	53 (19%)	23 (17%)	30 (22%)	0.298
**HDFs: diastole**				
A-B (%) (RMS)	8.0 (5.5, 10.9)	8.3 (6.5, 11.6)	7.2 (5.0, 9.8)	0.011
L-S (%) (RMS)	2.7 (1.9, 3.7)	2.9 (1.9, 3.9)	2.6 (1.8, 3.4)	0.098
L-S/A-B HDFs ratio (%)	34.9 (25.8, 44.5)	33.8 (25.2, 45.0)	36.8 (26.1, 44.5)	0.260
Angle φ (°)	68 (63, 72)	68 (63, 73)	67 (63, 71)	0.071

Abbreviations: CMR, cardiac magnetic resonance; STEMI, ST-segment elevation myocardial infarction; GLS, global longitudinal strain; GCS, global circumferential strain; EDV, end-diastolic volume; ESV, end-systolic volume; LVEF, left ventricular ejection fraction; IS, infarct size; MVO, microvascular obstruction; HDFs, hemodynamic forces; RMS, root mean square; A-B, apex–base; and L-S, latero-septal.

**Table 4:** Logistic regression analysis of factors associated with force reversal in STEMI patients.

Variable	Univariable		Stepwise Multivariable			
	OR (95% CI)	*p* Value	OR (95% CI)	*p* Value	OR (95% CI)	*p* Value
Anterior STEMI	2.27 (1.20–4.29)	0.011	2.22 (1.02–4.82)	0.044	2.31 (1.05–5.07)	0.036
GLS	1.05 (1.00–1.10)	0.03	0.98 (0.90–1.07)	0.791	0.98 (0.91–1.06)	0.772
GCS †	1.04 (1.00–1.08)	0.03	n.a.		1.03 (0.97–1.09)	0.245
LVEF †	0.97 (0.95–0.99)	0.028	0.98 (0.94–1.02)	0.363	n.a.	
IS	1.01 (0.99–1.03)	0.065	0.99 (0.97–1.02)	0.849	0.99 (0.97–1.02)	0.798
MVO	1.06 (1.00–1.12)	0.023	1.05 (0.98–1.13)	0.146	1.05 (0.98–1.13)	0.154

Abbreviations: † To avoid collinearity, GCS and LVEF were tested separately at multivariable logistic regression. STEMI, ST-segment elevation myocardial infarction; GLS, global longitudinal strain; GCS, global circumferential strain; LVEF, left ventricular ejection fraction; IS, infarct size; MVO, microvascular obstruction; OR, odds ratio; and CI, confidence interval.

**Table 5:** Univariable and multivariable Cox proportional hazard models for predicting heart failure.

Variables	Univariable		Stepwise Multivariable			
	HR (95% CI)	*p* Value	HR (95% CI)	*p* Value	HR (95% CI)	*p* Value
Force reversal	2.10 (1.22–3.62)	0.007	—		—	
Anterior STEMI	3.47 (1.91–6.31)	<0.001	—		—	
GCS †	1.09 (1.05–1.12)	<0.001	n.a.		—	
GLS	1.12 (1.09–1.15)	<0.001	1.09 (1.06–1.13)	<0.001	1.09 (1.06–1.13)	<0.001
LVEF †	0.94 (0.92–0.96)	<0.001	—		n.a.	
IS	1.05 (1.03–1.06)	<0.001	1.03 (1.02–1.05)	<0.001	1.03 (1.02–1.05)	0.004
MVO	1.08 (1.04–1.12)	<0.001	—		—	

Abbreviations: † To avoid collinearity, GCS and LVEF were tested separately at multivariable Cox regression. STEMI, ST-segment elevation myocardial infarction; GLS, global longitudinal strain; GCS, global circumferential strain; LVEF, left ventricular ejection fraction; IS, infarct size; MVO, microvascular obstruction; HR, hazard ratio; and CI, confidence interval.

**Table 6:** C-Statistics: Additive prognostic value of force reversal for heart failure.

	C-Statistic (95% CI)	*p* Value
Model 1 (LVEF)	0.680 (0.610–0.747)	—
Model 2 (IS)	0.717 (0.649–0.786)	—
Model 3 (GLS)	0.777 (0.726–0.827)	—
Model 4 (Anterior STEMI)	0.641 (0.584–0.697)	—
Model 5 (Force reversal)	0.626 (0.546–0.686)	—
Model 6 (Force reversal + LVEF)	0.770 (0.721–0.819)	0.034 Model 6 vs. Model 1
Model 7 (Force reversal + IS)	0.760 (0.697–0.823)	0.367 Model 7 vs. Model 2
Model 8 (Force reversal + GLS)	0.816 (0.775–0.858)	0.237 Model 8 vs. Model 3
Model 9 (Force reversal + Anterior STEMI)	0.702 (0.641–0.764)	0.146 Model 9 vs. Model 4

Abbreviations: STEMI, ST-segment elevation myocardial infarction; GLS, global longitudinal strain; LVEF, left ventricular ejection fraction; IS, infarct size; and CI, confidence interval.

## Data Availability

The data underlying this article will be shared upon reasonable request to the corresponding author.

## Supplementary Materials

The following supporting information can be downloaded at https://www.mdpi.com/article/10.3390/jcm14227978/s1; Table S1: Spearman’s correlation coefficients between hemodynamic force parameters and infarct size; Table S2: Intra-and inter-observer agreement for hemodynamic force measurements; Table S3: Assessment of multicollinearity for variables in the multivariable logistic regression model for force reversal.

## Abbreviations

The following abbreviations are used in this manuscript:

STEMI	ST-segment elevation myocardial infarction
CMR	Cardiac magnetic resonance
LVEF	Left ventricular ejection fraction
IVPGs	Intraventricular pressure gradients
HDFs	Hemodynamic forces
LGE	Late gadolinium enhancement
EDV	End-diastolic volume
ESV	End-systolic volume
IS	Infarct size
MVO	Microvascular obstruction
GLS	Global longitudinal strain
GCS	Global circumferential strain
PCI	Percutaneous coronary intervention
ECG	Electrocardiogram
bSSFP	Balanced steady-state free precession
A–B	Apical–basal
L–S	Lateral–septal
RMS	Root mean square
SD	Standard deviation
IQR	Interquartile range
ICC	Intraclass correlation coefficient
VIF	Variance inflation factor
CI	Confidence intervals
