# Supplementary material for: Force Reversal During Systolic–Diastolic Transition Provides Incremental Prognostic Value over LVEF for Heart Failure After STEMI
Source: J Clin Med. 2025 Nov 11;14(22):7978. doi: 10.3390/jcm14227978 (PMC12653228; doi:10.3390/jcm14227978)
Supplement: Supplementary file 1 [file jcm-14-07978-s001.zip › jcm-3939864-supplementary.pdf]

Table S1: Assessment of multicollinearity for variables in the multivariable logistic regression model for force reversal

**Variable**	VIF	Tolerance
Anterior STEMI	1.500	0.666
GLS	3.204	0.312
GCS	13.298	0.075
LVEF	17.238	0.058
IS	1.727	0.579
MVO	1.499	0.667

A VIF value < 5 was considered to indicate no significant multicollinearity. GCS and LVEF exhibited a high correlation (VIF > 10 when included in the same model) and were therefore tested in separate regression models.

STEMI, ST-segment elevation myocardial infarction; GLS, global longitudinal strain; GCS, global circumferential strain; LVEF, left ventricular ejection fraction; IS, infarct size; MVO, microvascular obstruction; VIF,variance inflation factor.

Table S2 Spearman's correlation coefficients between hemodynamic forces parameters and infarct size

Parameter	IS
**HDFs：entire heart cycle**	
A-B (%) (RMS)	-0.23*
L-S (%) (RMS)	-0.07
L-S / A-B HDFs ratio (%)	0.24*
Angle φ (°)	-0.29*
**HDFs: systole**	
A-B (%) (RMS)	-0.24*
L-S (%) (RMS)	-0.02
L-S/A-B HDFs ratio (%)	0.25*
Angle φ (°)	-0.23*
Systolic impulse (%)	-0.27*
Systolic peak (%)	-0.25*
**HDFs: sys-diastolic transition**	
A-B (%) (RMS)	-0.23*
L-S (%) (RMS)	-0.18*
L-S/A-B HDFs ratio (%)	0.09
Angle φ (°)	-0.08
LV suction (%)	0.24*
**HDFs: diastole**	
A-B (%) (RMS)	-0.22*
L-S (%) (RMS)	-0.06
L-S/A-B HDFs ratio (%)	0.18*
Angle φ (°)	-0.23*

* At the 0.05 scale (two-tailed), the correlation is significant.

HDFs, hemodynamic forces; RMS, root mean square; A-B, apex-base; L-S, latero-septal; IS, infarct size.

Table S3: Intra- and inter-observer agreement for hemodynamic forces measurements

Parameters	Inter-observer agreement	P value	Intra-observer agreement	P value
**HDFs: entire heart cycle**				
A-B (%) (RMS)	0.93 (0.89-0.99)	0.001	0.96 (0.91-0.99)	0.001
L-S (%) (RMS)	0.91 (0.86-0.97)	0.003	0.90 (0.86-0.97)	0.001
L-S/A-B HDF ratio (%)	0.89 (0.82-0.97)	0.001	0.86 (0.70-0.98)	0.002
Angle φ, (°)	0.93 (0.85-0.98)	0.001	0.91 (0.73-0.97)	0.001
**HDFs: systole**				
A-B (%) (RMS)	0.92 (0.80-0.98)	0.001	0.95 (0.83-0.99)	0.001
L-S (%) (RMS)	0.82 (0.46-0.95)	0.012	0.85 (0.54-0.91)	0.002
L-S/A-B HDF ratio (%)	0.94 (0.80-0.97)	0.001	0.82 (0.33-0.95)	0.02
Angle φ, (°)	0.87 (0.40-0.94)	0.001	0.81 (0.31-0.93)	0.004
Systolic impulse (%)	0.85 (0.37–0.93)	0.001	0.87 (0.42–0.94)	0.015
Systolic peak (%)	0.83 (0.28–0.93)	0.001	0.92 (0.84–0.98)	0.003
**HDFs: sys-diastolic transition**				
A-B (%) (RMS)	0.95 (0.86-0.97)	0.001	0.91 (0.82-0.96)	0.001
L-S (%) (RMS)	0.78 (0.36-0.93)	0.003	0.81(0.44-0.94)	0.014
L-S / A-B HDFs ratio (%)	0.78 (0.27-0.90)	0.027	0.89 (0.56-0.96)	0.006
Angle φ, (°)	0.83 (0.57-0.93)	0.001	0.84 (0.61-0.97)	0.013
LV suction (%)	0.79 (0.46-0.92)	0.016	0.82 (0.53-0.96)	0.007
Force reversal (%)	0.82 (0.60-0.94)	0.012	0.85 (0.65-0.96)	0.006
**HDFs: diastole**				
A-B (%) (RMS)	0.96 (0.90-0.98)	0.001	0.94 (0.89-0.98)	0.001
L-S (%) (RMS)	0.79 (0.24-0.92)	0.017	0.83 (0.37-0.95)	0.005
L-S/A-B HDF ratio (%)	0.78 (0.45-0.95)	0.032	0.90 (0.61-0.97)	0.021
Angle φ , (°)	0.88 (0.41-0.97)	0.001	0.93 (0.68-0.97)	0.001

HDFs, hemodynamic forces; RMS, root mean square; A-B, apex-base; L-S, latero-septal

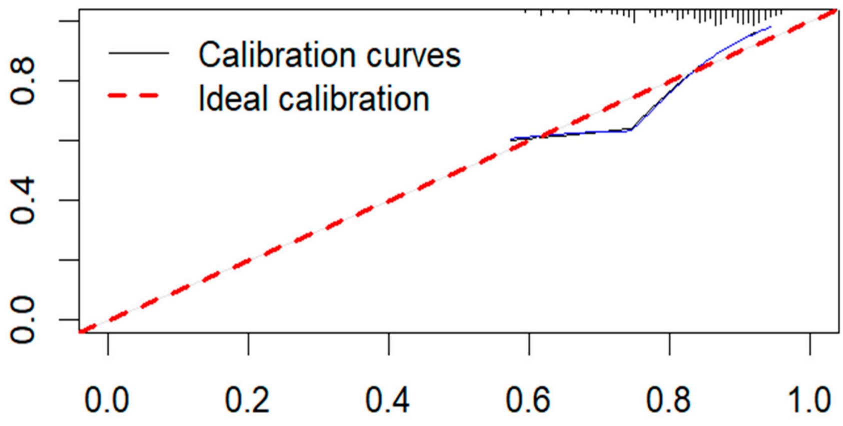

Figure S1. The calibration curve of Model 6 for predicting HF with a follow-up of 1 year
